# Shared genetic influences between dimensional ASD and ADHD symptoms during child and adolescent development

**DOI:** 10.1186/s13229-017-0131-2

**Published:** 2017-04-04

**Authors:** Evie Stergiakouli, George Davey Smith, Joanna Martin, David H. Skuse, Wolfgang Viechtbauer, Susan M. Ring, Angelica Ronald, David E. Evans, Simon E. Fisher, Anita Thapar, Beate St Pourcain

**Affiliations:** 1 MRC Integrative Epidemiology Unit (MRC IEU), University of Bristol, Bristol, UK; 2 School of Oral and Dental Sciences, University of Bristol, Bristol, UK; 3 School of Social and Community Medicine, University of Bristol, Bristol, UK; 4 Stanley Center for Psychiatric Research, Broad Institute of MIT and Harvard, Cambridge, MA USA; 5 Department of Medical Epidemiology and Biostatistics, Karolinska Institute, Stockholm, Sweden; 6 MRC Centre for Neuropsychiatric Genetics and Genomics, Cardiff University, Cardiff, UK; 7 Institute of Child Health, University College London, London, UK; 8 Department of Psychiatry and Neuropsychology, Maastricht University, Maastricht, The Netherlands; 9 Department of Psychological Sciences, Birkbeck, University of London, London, UK; 10 University of Queensland Diamantina Institute, Translational Research Institute, University of Queensland, Brisbane, Australia; 11 Language and Genetics Department, Max Planck Institute for Psycholinguistics, Nijmegen, The Netherlands; 12 Donders Institute for Brain, Cognition and Behaviour, Radboud University, Nijmegen, The Netherlands

**Keywords:** Social communication, ADHD symptoms, Clinical ADHD, ALSPAC, Genetic overlap

## Abstract

**Background:**

Shared genetic influences between attention-deficit/hyperactivity disorder (ADHD) symptoms and autism spectrum disorder (ASD) symptoms have been reported. Cross-trait genetic relationships are, however, subject to dynamic changes during development. We investigated the continuity of genetic overlap between ASD and ADHD symptoms in a general population sample during childhood and adolescence. We also studied uni- and cross-dimensional trait-disorder links with respect to genetic ADHD and ASD risk.

**Methods:**

Social-communication difficulties (*N* ≤ 5551, Social and Communication Disorders Checklist, SCDC) and combined hyperactive-impulsive/inattentive ADHD symptoms (*N* ≤ 5678, Strengths and Difficulties Questionnaire, SDQ-ADHD) were repeatedly measured in a UK birth cohort (ALSPAC, age 7 to 17 years). Genome-wide summary statistics on clinical ASD (5305 cases; 5305 pseudo-controls) and ADHD (4163 cases; 12,040 controls/pseudo-controls) were available from the Psychiatric Genomics Consortium. Genetic trait variances and genetic overlap between phenotypes were estimated using genome-wide data.

**Results:**

In the general population, genetic influences for SCDC and SDQ-ADHD scores were shared throughout development. Genetic correlations across traits reached a similar strength and magnitude (cross-trait *r*_g_ ≤ 1, *p*_min_ *=* 3 × 10^−4^) as those between repeated measures of the same trait (within-trait *r*_g_ ≤ 0.94, *p*_min_ *=* 7 × 10^−4^). Shared genetic influences between traits, especially during later adolescence, may implicate variants in K-RAS signalling upregulated genes (*p*-meta = 6.4 × 10^−4^).

Uni-dimensionally, each population-based trait mapped to the expected behavioural continuum: risk-increasing alleles for clinical ADHD were persistently associated with SDQ-ADHD scores throughout development (marginal regression *R* ^2^ = 0.084%). An age-specific genetic overlap between clinical ASD and social-communication difficulties during childhood was also shown, as per previous reports. Cross-dimensionally, however, neither SCDC nor SDQ-ADHD scores were linked to genetic risk for disorder.

**Conclusions:**

In the general population, genetic aetiologies between social-communication difficulties and ADHD symptoms are shared throughout child and adolescent development and may implicate similar biological pathways that co-vary during development. Within both the ASD and the ADHD dimension, population-based traits are also linked to clinical disorder, although much larger clinical discovery samples are required to reliably detect cross-dimensional trait-disorder relationships.

**Electronic supplementary material:**

The online version of this article (doi:10.1186/s13229-017-0131-2) contains supplementary material, which is available to authorized users.

## Background

An aetiological link between attention-deficit/hyperactivity disorder (ADHD) symptoms and autism spectrum disorder (ASD) symptoms has been supported by family [1, 2] and twin studies [3–7], and shared genetic influences [8, 9] have been reported both throughout population variation [3–7, 10] and at the extreme [5, 11]. Clinical ADHD is a common childhood disorder with a prevalence around 3.4% [12] and characterised by hyperactive-impulsive and inattentive behavioural symptoms. According to clinical classification systems, the age of onset for ADHD has been defined before 7 years [13] and recently changed to 12 years in DSM-5 [14]. ASD has a typical age of onset before the age of 3 years [13], affecting ~1 to 2% of children [15, 16]. Core features include deficits in social interaction and communication, as well as highly restricted interests and/or stereotyped repetitive behaviours [13]. The underlying genetic aetiology of both ADHD and ASD is complex, with contributions of both rare and common variation [17–20] (where the latter are defined throughout this paper as variants with a minor allele frequency of ≥1%).

Although twin and family studies suggest that ADHD and ASD symptoms are co-heritable [8, 9], studies investigating molecular genetic links between clinical ADHD and clinical ASD have provided mixed support for the hypothesis of shared aetiologies between both conditions. Copy number variation (CNV) analyses have identified shared biological pathways in clinical ADHD and ASD [21]. Analyses using genome-wide array data [22, 23] have reported, in contrast, little evidence for genetic overlap between both conditions, probably as a consequence of limited power to date.

Beyond the concept of dichotomous entities, common disorders such as clinical ASD and ADHD can be understood as extreme values on one or more continuous underlying scales of liability [24], due to their polygenic architecture. These views are consistent with theories conceptualising ADHD and ASD as upper extremes of an underlying behavioural continuum [25–27], implicating a uni-dimensional trait-disorder overlap. Studies of social-communication difficulties assessed in children from the general population and samples of clinical ASD have recently identified shared genetic links using genome-wide summary data [28]. Similarly, population-based ADHD symptoms, when measured during childhood, share genetic links with clinically diagnosed ADHD, as captured by common polygenic risk [29, 30]. Thus, it is conceivable to ask whether there exist similar pleiotropic effects between traits and clinical disorders across behavioural dimensions, implicating links between ADHD symptoms and clinical ASD and, equivalently, between ASD symptoms and clinical ADHD, i.e. a cross-dimensional trait-disorder overlap. However, studies have demonstrated developmental heterogeneity in the genetic overlap between ASD and ADHD symptoms, especially in non-clinical populations [31]. Twin studies reported low genetic correlations during infancy [32] that rise to moderate strength during childhood and adolescence [31] and remain moderate to strong in adults [3, 6]. These findings concur with studies reporting developmental changes within the genetic architecture of both, ASD and ADHD symptoms [31]. The contribution of genetic factors to ASD and ADHD symptom overlap during development, as tagged by common genetic variation, is, however, largely unexplored. Recent research started investigating the association between risk-increasing alleles for clinical ADHD and communication problems in children from the general population [29]. Comparatively, little is known, however, of cross-trait genetic relationships during child and adolescent development and whether cross-dimensional trait-disorder relationships are developmentally sensitive with respect to the age of the population-based trait.

The aim of this work is to provide insight into the genetic overlap across ADHD- and ASD-related dimensions during the course of child and adolescent development. For this, we investigate a phenotypically rich longitudinal population-based cohort from the UK, the Avon Longitudinal Study of Parents and Children (ALSPAC), as well as summary statistics from the largest publicly available clinical ADHD and ASD samples collected by the Psychiatric Genomics Consortium (PGC) [22, 33]. Given the strong genetic overlap between communication difficulties and ADHD traits in community twin samples [34], we selected social-communication difficulties as well as combined hyperactive-impulsive and inattentive ADHD symptoms for the study on the population level. Here, we (a) report and characterise genetic links between longitudinally assessed social-communication difficulties and combined hyperactive-impulsive and inattentive ADHD symptoms within the general population across ages 7 to 17 years, (b) confirm that these traits genetically overlap with clinical disorder assuming a uni-dimensional behavioural continuum (unless already reported) and (c) study the cross-dimensional trait-disorder overlap between these longitudinally assessed population-based traits with respect to both clinical ADHD and ASD.

## Methods

### Population-based samples and measures

Population-based analyses were performed in ALSPAC children, a UK population-based longitudinal pregnancy-ascertained birth cohort (estimated birth date: 1991 to 1992) [35]. Please note that the study website contains details of all the data that is available through a fully searchable data dictionary [36] and that data access can be requested through the ALSPAC Executive Committee.

#### ADHD symptoms

The Strengths and Difficulties Questionnaire (SDQ) [37] is a behavioural screening instrument with high reliability and validity with respect to the identification of a psychiatric diagnosis [38]. A subscale of the SDQ assesses combined hyperactive-impulsive and inattentive ADHD symptoms using five items (three hyperactive-impulsive and two inattentive items): ‘Restless, overactive. cannot stay still for long’ (hyperactive-impulsive), ‘Constantly fidgeting or squirming’ (hyperactive-impulsive), ‘Easily distracted, concentration wanders’ (inattentive), ‘Thinks things out before acting’ (hyperactive-impulsive, reverse coded) and ‘Sees tasks through to the end. Good attention span’ (inattentive, reverse coded). These items are rated as ‘Not True’ (0), ‘Somewhat true’ (1) and ‘Certainly true’ (2) and combined into summary score, with higher scores indicating more behavioural problems (range 0–10). These items load primarily on their intended factor, i.e. hyperactivity-impulsivity/inattention, and show minimal cross-loadings on other factors assessed by the SDQ [37, 39]. Mother-reports on their children’s hyperactivity-impulsivity and inattention were obtained at 7, 10, 12, 13 and 17 years of age (referred to as SDQ-ADHD subscale in this study). An additional SDQ-ADHD measure at 8 years of age was excluded due to potential bias (Additional file 1: Additional note). Information on phenotypic and genotypic data was available for 4164 to 5612 children (Table 1, Additional file 1: Table S1).

**Table 1:** Population-based and clinical samples

Sample	Source	Ethnicity	Number	Phenotype/diagnosis
ALSPAC^a,b^	General population	White European	5612 (7 y): age(SE) = 6.79(0.11)	ADHD symptoms (mother-report) as assessed with SDQ ADHD scores
			5678 (10 y): age(SE) = 9.65(0.12)	
			5259 (12 y): age(SE) = 11.72(0.13)	
			5072 (13 y): age(SE) = 13.16(0.18)	
			4164 (17 y): age(SE) = 16.84(0.18)	
ALSPAC^a,b^	General population	White European	5551 (8 y): age(SE) = 7.65(0.14)	Social-communication difficulties (mother-report) as assessed with SCDC scores
			5460 (11 y): age(SE) = 10.72(0.13)	
			5060 (14 y): age(SE) = 13.90(0.15)	
			4174 (17 y): age (SE) = 16.84(0.36)	
PGC-ADHD^c^	Clinical ADHD sample	White European	4163 cases and 12,040 controls/pseudo-controls	ADHD or hyperkinetic disorder
PGC-ASD^a^	Clinical ASD sample	White European	5305 cases; 5305 pseudo-controls	ASD

*ADHD* attention deficit hyperactivity disorder, *ALSPAC* Avon Longitudinal study of Parents and Children, *ASD* autism spectrum disorder, *PGC-ADHD* ADHD collection of the Psychiatric Genomics Consortium (PGC), *PGC-ASD* ASD collection of the PGC, *SCDC* Social and Communication Disorders Checklist, *SDQ-ADHD* ADHD subscale of the Strength and Difficulties Questionnaire, *y* age in years

^a^Samples were imputed to a 1000 genomes reference (Phase1_v3)

^b^ALSPAC individuals who were related to participants of the PGC-ADHD sample were excluded

^c^Samples were imputed to HapMap3 CEU and TSI

#### Social-communication difficulties

Quantitative social-communication difficulties in ALSPAC participants were measured with the 12-item Social and Communication Disorders Checklist (SCDC; score range 0 to 24) [40]. The SCDC is a brief screening instrument of social reciprocity and verbal/nonverbal communication (e.g. ‘Not aware of other people’s feelings’). It has high reliability and internal consistency and good discriminant validity between pervasive developmental disorder and other clinical groups [40] with higher scores reflecting more social-communication deficits (positively skewed). There is substantial trait overlap between the SCDC items and canonical ASD symptomology, and children with ASD have high average scores on the SCDC [40]. Mother-reported SCDC scores for children and adolescents, using the full scale, were repeatedly measured at 8, 11, 14 and 17 years, and information on phenotypic and genotypic data was available for 4174 to 5551 children (Table 1, Additional file 1: Table S1).

#### Phenotype transformations

Descriptive analyses in R (R.v.3.2.4) showed that untransformed scores for both traits were positively skewed and predominantly leptokurtic, especially for SCDC scores (Additional file 1: Table S1), as previously reported [41]. All scores were therefore transformed as some of the methods used in this study assume multivariate normality (see below). Specifically, scores were adjusted for sex, age and the two most significant ancestry-informative principal components (see below) using ordinary least square (OLS) regression, and residuals were subsequently rank-transformed to normality. Within-trait phenotypic correlations for both, the SDQ-ADHD subscale and the SCDC, showed phenotypic stability during development, with stronger correlations across narrower age gaps and weaker correlations across wider age gaps. Correlation estimates for both untransformed (based on Spearman’s rank correlation) and transformed (based on Pearson product moment correlation) trait scores were highly similar (SDQ-ADHD, Additional file 1: Table S2: Spearman’s *ρ* = 0.46 to 0.70; Pearson *r* = 0.46 to 0.69; SCDC, Additional file 1: Table S3: Spearman’s *ρ* = 0.39 to 0.57; Pearson *r* = 0.38 to 0.61), as previously reported for the SCDC [41, 42].

Phenotypic correlations (*r*_p_) across traits were estimated using Pearson product moment correlation coefficients based on rank-transformed traits. To account for the multiple testing burden within this study, we estimated the effective number of independent population-based phenotypes studied within this work based on the eigenvalues of the correlation matrix of the assessed SCDC and SDQ-ADHD scores [43]. This revealed five independent measures corresponding to an experiment-wise error rate of 0.0102. We excluded SCDC scores at 14 years from this calculation, as this measure has very low single-nucleotide polymorphism heritability (SNP-h^2^), as previously reported [42] (shown for completeness only). The applied experiment-wise error rate is likely to be conservative as longitudinal patterns are ignored and measures with low SNP-h^2^ will contribute little to genetic cross-trait links. Therefore, both unadjusted and adjusted findings are reported.

#### Genome-wide data

ALSPAC children were genotyped using the Illumina HumanHap550 quad chip genotyping platforms. The ALSPAC genome-wide association study (GWAS) data was generated by Sample Logistics and Genotyping Facilities at the Wellcome Trust Sanger Institute and Laboratory Corporation of America using support from 23andMe. After quality control (individual call rate >0.97, SNP call rate >0.95, Minor allele frequency (MAF) >0.01, Hardy-Weinberg equilibrium (HWE) *p* > 10^−7^ and removal of individuals with cryptic relatedness and non-European ancestry), 8237 children and 477,482 directly genotyped SNPs were retained. SNPs were flipped to the forward strand and haplotypes were estimated with ShapeIT (v2.r644) [44]. Pertinent to this study, two ALSPAC children who were related to participants of the PGC ADHD sample at the second cousin level (or closer) were also excluded (based on their genetic relationship). Imputation was performed using Impute V2.2.2 [45] in 1000 genomes reference haplotypes (Phase1_v3 [46]).

### Genome-wide summary information on clinical ADHD

The publically available results from an internationally collaborative mega-analysis of clinical ADHD, conducted by the Psychiatric Genomics Consortium (PGC), were utilised in this study. This sample consisted of 4163 cases and 12,040 controls/pseudo-controls of predominantly European descent [22, 33]. ADHD cases were aged between 5 and 17 years and met diagnostic criteria for either clinical ADHD or hyperkinetic disorder based on either the Diagnostic and Statistical Manual of Mental Disorders (DSM-III, DSM-IV, DSM-TR) or the International Classification of Diseases (ICD-10) [33]. Genome-wide data for this mega-analysis were imputed to a HapMap Phase III European CEU (HapMap3 CEU) and TSI (HapMap3 TSI) panel.

### Genome-wide summary information on clinical ASD

A genome-wide scan of 5305 ASD cases and their parents (PGC-ASD), all of European ancestry (2015 freeze), has been completed by the PGC. Cases obtained an ASD diagnosis using research standard diagnoses and expert clinical consensus diagnoses at the age of 3 years or above. More than 90% of all patients had also a diagnosis of autism from the Autism Diagnostic Interview-Revised [47] and/or the Autism Diagnostic Observation Schedule [48]. The analyses were conducted using a case and pseudo-control design [49], which is robust to population stratification, as cases and pseudo-controls are ancestrally matched.

There is no sample overlap between population-based and clinical samples (Table 1).

### Genetic-relationship-matrix restricted maximum likelihood estimation

Longitudinally assessed SCDC scores and SDQ-ADHD scores were studied using genetic-relationship-matrix restricted maximum likelihood (GREML) to estimate the proportion of phenotypic variation due to genetic factors (genetic variance, Var_g_), as tagged by common SNPs on a genotyping chip. Analyses were carried out with Genome-wide Complex Trait Analysis (GCTA) software [50]. For rank-transformed traits, estimates of Var_g_ are equivalent to estimates of SNP-h^2^, as the phenotypic variance has been standardised to one. Genetic relationship matrices (GRMs) were constructed using directly genotyped SNPs, excluding individuals with a pairwise relationship >0.025 [50].

Bivariate GREML [51] was carried out to estimate genetic correlations (*r*_g_) that capture the extent to which two phenotypes share genetic factors (ranging between −1 and 1), as tagged by common variation. In addition, we report genetic covariances (Cov_g_). These estimates represent, for rank-transformed traits [52], the part of the phenotypic correlation that can be explained by genetic factors. Note that for rank-transformed traits, the genetic covariance is equivalent to the product of the square root of the heritabilities of the two phenotypes and their genetic correlation [52]. Genetic relationships were analysed between longitudinally assessed measures of the same trait (‘within-trait’, i.e. for SCDC and SDQ-ADHD scores separately) and across traits (‘cross-trait’, i.e. between SCDC and SDQ-ADHD scores). In addition, we estimated the residual correlations (*r*_e_) between measures. Within-trait analyses for SCDC scores correspond to previously published findings [42] and are reported for comparison only. All analyses were performed using rank-transformed residuals in order to adhere to GREML multivariate normality assumptions. Note that bivariate GREML using untransformed scores (adjusted for covariates) resulted in slow or no convergence.

### Pathway analysis

We used the molecular signatures database (MSigDB) hallmark gene set collection [53] to dissect the genetic variance of population-based traits that were assessed at different stages during development (four SCDC score measures at 8, 11, 14 and 17 years and five SDQ-ADHD score measures at 7, 10 12, 13 and 17 years). The gene sets were derived from multiple ‘founder’ MSigDB gene sets and represent a specific biological state or process with coherent expression and little redundancy [53]. For each of the 50 biological pathways, we selected genetic variation from each member of the gene set (±50 kb flanking each gene boundary, hg19, autosomal genes only) and constructed two sets of GRMs, using PLINKv1.9 [54] and GCTA software [50]. The first GRM contained all SNPs within the gene set (set-GRM), and the second GRM contained the remaining SNPs (noset-GRM). Using GREML, we compared full models (set-GRM and noset-GRM) with reduced models (noset-GRM only) using likelihood ratio tests (LRTs). For each pathway, the statistical evidence across both traits (four SCDC scores at 8, 11, 14 and 17 years and five SDQ-ADHD scores at 7, 10 12, 13 and 17 years) was finally combined using a homogeneity statistic [55], accounting for the genetic overlap among measures (Additional file 1: Additional note). All pathway-specific meta-analysis statistics were corrected for 50 independent tests using a Bonferroni threshold. To allow for the possibility that some signals are driven by the number of SNPs included in the GRM, which is approximately proportional to gene size, we also carried out permutation analyses. We randomly generated 200 sets of genes with similar sizes as observed for the selected pathway to create a null distribution of test statistics, and derived empirical *p* values. In addition, we studied the presence of age-specific and trait-specific changes in explained genetic variance using a random effects meta-regression approach (R:metafor), accounting for two interrelated traits (four SCDC score measures at 8, 11, 14 and 17 years and five SDQ-ADHD score measures at 7, 10 12, 13 and 17 years) and assuming multivariate normality. In addition to a random intercept, the model included fixed effects for age at assessment and fixed effects for trait (SCDC scores coded as zero and ADHD-SDQ scores coded as one). Beta coefficients measure here the increase in variance per one year as well as the difference in explained variance between the two traits. A variance/covariance matrix across the nine measures was approximated analogous to models accounting for correlated phylogenetic histories [56] including the observed phenotypic correlation matrix between the nine measures weighted by the standard errors of the genetic variance as estimated by GREML. We also examined the evidence for a fixed trait × age interaction effect using LRTs.

### Polygenic scoring analyses

Polygenic risk scores (PGS) [57, 58] were generated in ALSPAC using PGC summary statistics. Using a range of *p* value thresholds (0.001 < *P*_*T*_ ≤ 1), PGS for ASD (based on PGC-ASD) and ADHD (based on PGC-ADHD) were constructed in ALSPAC (Table 1) using imputed genotypes (1000 Genomes, PhaseI_v3, INFO >0.8). Autosomal PGC-ASD and PGC-ADHD GWAS signals with MAF of above 0.01 in ALSPAC were clumped (linkage disequilibrium *r*_2_ > 0.25, ±500 kb) according to current guidelines [59] using PLINK [54] software and excluding duplicate SNPs. For age-specific analyses, rank-transformed population-based traits were regressed on Z-standardised PGS using ordinary least square regression (Rv3.2.2). The proportion of phenotypic variance explained by each PGS predictor is reported as adjusted regression *R* ^2^. Longitudinal analyses were carried out using a mixed effects regression modelling framework (R:lme4). Repeatedly measured untransformed SDQ scores were regressed on PGS including fixed effects for PGS, sex and age at assessment, and random slopes (for age) and intercepts. Beta coefficients for PGS quantify here the increase in trait scores for each increase in one standard deviation of PGS. Differences in sample dropout across time were accounted for through bootstrapping, and we generated parametric 95% bootstrap confidence intervals (95% CI_Bootstrap_ with N_Bootstrap_ = 500). In the presence of association, we estimated the marginal regression *R* ^2^ [60] (R:MuMIn, linear mixed effects models only). Repeatedly measured untransformed SCDC scores were analysed similarly, but using a mixed Poisson regression framework and random intercepts only. Beta coefficients for PGS quantify in this model the increase in natural-log scores for each increase in one standard deviation of PGS. Statistical significance for all longitudinal analyses was assessed using likelihood ratio tests. We finally estimated the expected genetic covariance between trait and disorder using the Avengeme software [58, 61], based on findings from polygenic scoring. We assumed for simplicity, 100,000 causal SNPs for each disorder, and a complex architecture as described in a recent PGC study [22] (PGC-ASD: prevalence = 0.01, liability-scale SNP-h^2^ = 0.17; PGC-ADHD: prevalence = 0.05, liability-scale SNP-h^2^ = 0.28).

## Results

### Genetic variance of traits using longitudinally assessed measures

**Fig. 1:**
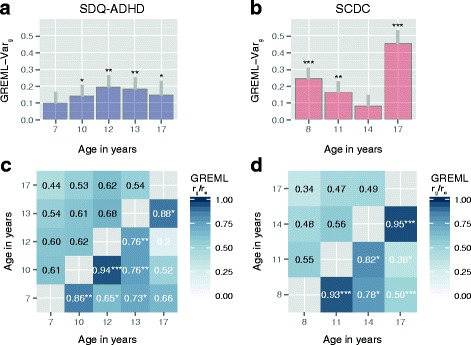
Genetic architecture of SDQ-ADHD and SCDC scores. Genetic-relationship-matrix restricted maximum likelihood (GREML) genetic variance (Var_g_), genetic (*r*_g_) and residual correlations (*r*_e_) are shown for SDQ-ADHD scores (**a**, **c**) and SCDC scores (**b**, **d**) in ALSPAC; *grey bars* (**a**, **b**) indicate one GREML-h^2^ standard error; *r*_g_ estimates for each trait (**b**, **d**) are shown in the *lower triangle*, *r*_e_ estimates (**b**, **d**) in the *upper triangle*. *ALSPAC* Avon Longitudinal Study of Parents and Children, *SCDC* Social and Communication Disorders Checklist at 8, 11, 14 and 17 years (rank-transformed), *SDQ-ADHD* ADHD subscale of the Strength and Difficulties Questionnaire at 7, 10 12, 13 and 17 years (rank-transformed); note that for rank-transformed traits, estimates of SNP-h^2^ are equivalent to estimates of Var_g_, as the phenotypic variance has been standardised to one. *r*_g_ *p* values: *p ≤ 0.05, **p ≤ 0.01 and ***p ≤ 0.001 (uncorrected for multiple testing, experiment-wise error rate *p* = 0.01)

Within ALSPAC, the strongest evidence for genetic effects contributing to SDQ-ADHD scores, as captured by common variants, was identified during late childhood and early adolescence (age 12: GREML-Var_g_(SE) = 0.19(0.07), *p* = 0.002; age 13: GREML-Var_g_(SE) = 0.18(0.07), *p* = 0.003; Fig. 1a, Additional file 1: Table S4). For SCDC scores, genetic effects were observed during early and middle childhood as well as later adolescence (age 8: GREML-Var_g_(SE) = 0.24(0.07), *p* = 7.0 × 10^−5^; age 11: GREML-Var_g_(SE) = 0.16(0.07), *p* = 0.005; age 17: GREML-Var_g_(SE) = 0.45(0.09), *p* = 3.0 × 10^−9^; Fig. 1b, Additional file 1: Table S5), with a drop during early adolescence (age 14: GREML-Var_g_(SE) = 0.08(0.07), *p* = 0.10), similar to previously reported findings on the SCDC [41, 42]. For both traits, estimates of heritability were nearly identical to genetic variance estimates (Additional file 1: Tables S4 and S5). For comparison only, we also studied untransformed scores, irrespective of violations of the assumption of normality, and found similar results, although the observed estimates were less strong (Additional file 1: Table S4 and S5).

### Within-trait genetic overlap using longitudinally assessed measures

In the ALSPAC sample, we found evidence for genetic links among repeatedly assessed SDQ-ADHD scores throughout development, as estimated with bivariate GREML. The genetic correlation was strongest for measures assessed at similar developmental stages (Fig. 1c, Additional file 1: Table S6), especially during childhood and early adolescence, and the magnitude of the correlation decreased with increasing age gaps. Similar correlation patterns were also observed for SCDC scores (Fig. 1d, Additional file 1: Table S7), as previously reported [42] (shown for completeness only).

### Cross-trait genetic overlap using longitudinally assessed measures

**Fig. 2:**
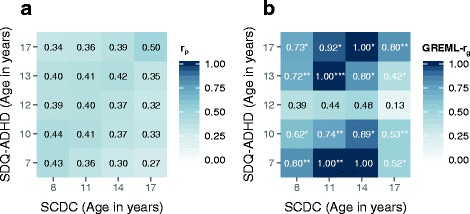
Cross-trait phenotypic (**a**) and genetic correlations (**b**) between SDQ-ADHD and SCDC scores during childhood and adolescence. Using the ALSPAC cohort, cross-trait phenotypic correlations (*r*_p_) were estimated using Pearson product moment correlation coefficients (*p* ≤ 0.001) and cross-trait genetic correlations (*r*_g_) were estimated with bivariate GREML. *ALSPAC* Avon Longitudinal Study of Parents and Children, *GREML* genetic-relationship-matrix restricted maximum likelihood, *GREML-r*_*g*_ bivariate genetic correlation, *SCDC* Social and Communication Disorders Checklist at 8, 11, 14 and 17 years (rank-transformed), *SDQ-ADHD* ADHD subscale of the Strength and Difficulties Questionnaire at 7, 10 12, 13 and 17 years (rank-transformed). *r*_g_ (*p* values): *p ≤ 0.05, **p ≤ 0.01 and ***p ≤ 0.001 (uncorrected for multiple testing, experiment-wise error rate *p* = 0.01)

Consistent with phenotypic cross-trait correlations (Fig. 2a, *r*_pheno_ *p* < 0.001, Additional file 1: Table S8) in ALSPAC, reaching moderate strength across similar age bands, genetic overlap between SDQ-ADHD and SCDC scores was identified throughout development (Fig. 2b, Additional file 1: Table S8). Of note, some cross-trait genetic correlations reached a similar magnitude and strength as correlations between measures of the same trait (e.g. SCDC_age11__SDQ-ADHD_age13_: GREML-*r*_g_(SE) = 1.00(0.26), *p* = 3 × 10^−4^ versus SDQ-ADHD _age10__SDQ-ADHD_age12_: GREML-*r*_g_(SE) = 0.94(0.13), *p* = 7 × 10^−4^). The respective genetic covariances were nearly identical (SCDC_age11__SDQ-ADHD_age13_: GREML-Cov_g_(SE) = 0.17(0.05) versus SDQ-ADHD_age10__SDQ-ADHD_age12_: GREML-Cov_g_(SE) = 0.17(0.06)). The relative contribution of genetic to phenotypic covariance (i.e. the co-heritability [62]) may, however, differ by trait (co-heritability SCDC_age11__SDQ-ADHD_age13_: ~41% based on *r*_p_ = 0.41, Additional file 1: Table S8; co-heritability SDQ-ADHD_age10__SDQ-ADHD_age12_: ~25% based on *r*_p_ = 0.67, Additional file 1: Table S2).

Genetic correlations between SDQ-ADHD and SCDC scores were, however, developmentally sensitive and dropped for cross-trait genetic links involving SDQ-ADHD scores during very early adolescence (~12 years of age), despite cross-trait phenotypic correlations (Fig. 2a). Note that SDQ-ADHD scores at 12 years are heritable (GREML-Var_g_ (SE) = 0.19(0.07), *p* = 0.002; Fig. 1a; Additional file 1: Table S4) and genetically related to SDQ-ADHD measures at other ages (Fig. 1c, Additional file 1: Table S6).

Given the evidence for persistent genetic cross-trait links throughout development, we investigated the hypothesis that phenotypic variation in both traits is influenced by similar biological pathways. As multivariate approaches to annotate the genetic covariance between phenotypes are currently not available yet, we dissected the genetic variance of SCDC scores and SDQ-ADHD scores (four SCDC score measures at 8, 11, 14 and 17 years and five SDQ-ADHD score measures at 7, 10 12, 13 and 17 years) according to MSigDB hallmark gene sets and then combined the evidence.

**Fig. 3:**
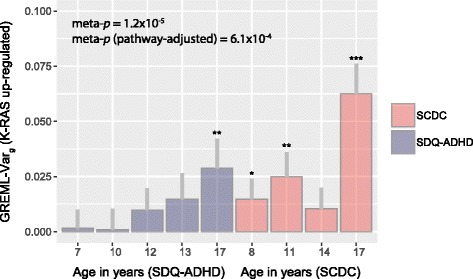
Genetic variance in SDQ-ADHD and SCDC scores due to variation within K-RAS signalling upregulated genes. The genetic variance composition for SCDC and SDQ-ADHD scores in ALSPAC was dissected according to genetic variation within molecular signatures database (MSigDB) hallmark gene set collections [53]. For each measure, the genetic variance is shown for the pathway of K-RAS signalling upregulated genes including one standard error (*grey bar*) and measurement-specific *p* values (*p ≤ 0.05, **p ≤ 0.01 and ***p ≤ 0.001). Statistical evidence across measures was combined using a homogeneity statistic [55], accounting for phenotypic overlap among traits. The meta-analysis *p* value (meta-p) reflects four SCDC scores (at 8, 11, 14 and 17 years) and five SDQ-ADHD scores (7, 10 12, 13 and 17 years) and is Bonferroni-adjusted for 50 analysed pathways (pathway-adjusted). *ALSPAC* Avon Longitudinal Study of Parents and Children, *SCDC* Social and Communication Disorders Checklist at 8, 11, 14 and 17 years (rank-transformed), *SDQ-ADHD* ADHD subscale of the Strength and Difficulties Questionnaire at 7, 10 12, 13 and 17 years (rank-transformed)

Among the 50 analysed gene sets, genetic variation within V-Ki-ras2 Kirsten rat sarcoma viral oncogene homolog (K-RAS) signalling upregulated genes accounted for genetic variance in both traits (*p*_meta_ = 0.000012; *p*_meta_ (pathway-adjusted) = 6.1 × 10^−4^; including all repeatedly assessed measures). The largest variance contributions were found in later adolescence, where K-RAS signalling upregulated genes explained nearly 3% in phenotypic variation in SDQ-ADHD scores and over 6% in SCDC scores (Fig. 3, Additional file 2: Table S9). We did not observe a similar joint signal using 200 randomly generated gene sets, with equivalent gene numbers and gene size as the K-RAS signalling upregulated gene set (empirical *p* < 0.005). Using a random effects meta-regression approach showed that the genetic variance accounted for by the K-RAS signalling upregulated gene set increases with progressing age (*β*_Age_(SE) = 0.0029(0.0013), *p* = 0.023), with little support for trait-specific effects (*β*_Trait_(SE) = −0.013(0.0093), *p* = 0.16) or trait-specific developmental changes (*β*_Trait × Age_ (SE) = -0.001(0.002), *p* = 0.67). There was also little evidence for the contribution of other pathways to shared phenotypic variation across traits, beyond chance (Additional file 2: Table S9).

### Genetic overlap between population-based traits and clinical disorder

To estimate the proportion of phenotypic variance in population-based traits due to risk increasing alleles contributing to liability for disorder, we also applied polygenic scoring.

*Uni-dimensional trait-disorder overlap*: We found evidence for uni-dimensional trait-disorder overlap between SDQ-ADHD scores and clinical ADHD throughout development. Age-specific association at the nominal level was observed for SDQ-ADHD measures at 7, 10, 13 and 17 years (Additional file 1: Table S10). Longitudinal modelling of untransformed SDQ-ADHD scores against polygenic risk scores suggested that the link between ADHD scores and clinical ADHD is persistent over time (for example at a PGS threshold *P*_*T*_ = 0.5: *β*_ADHD-PGS_(95% CI_Bootstrap_) = 0.067 (0.023, 0.11), *p* = 0.0043) with little evidence for developmental changes in effect (*β*_ADHD-PGS × age_ (95% CI_Bootstrap_) = 0.0008(−0.005; 0.0074); *p* = 0.80). The marginal adjusted *R* ^2^ in SDQ-ADHD scores due to ADHD-PGS at *P*_*T*_ = 0.5 was 0.084%, adjusted for age and sex, and the corresponding genetic covariance was estimated as 0.10 (95% CI 0.03, 0.17) (approximated SE = 0.035 assuming multivariate normality). An age-specific genetic link between clinical ASD and SCDC scores at age 8 (but not later during development) has been previously reported [28, 41].

*Cross-dimensional trait-disorder overlap*: We found little support that alleles more common in ASD cases than in controls/pseudo-controls are associated with variation in SDQ-ADHD scores, including both age-specific (Additional file 1: Table S11) and longitudinal models (for example at a PGS threshold *P*_*T*_ = 0.5: *β*_ASD-PGS_(95% CI_Bootstrap_) = −0.0034 (−0.051, 0.046), *p* = 0.32).

There was also little evidence that alleles more common in ADHD cases than in controls/pseudo-controls are robustly related to variation in SCDC scores at 8, 11, 14 or 17 years (Additional file 1: Table S12) when applying an experiment-wise error rate of 0.01. There was a trend for association with rank-transformed SCDC scores at age 14 years (for example at a PGS threshold of 0.5: *β*_ADHD-PGS_age14_(SE) = 0.028(0.01), *p* = 0.049). Longitudinal analyses of untransformed scores provided, however, little evidence for a consistent effect (for example at a PGS threshold *P*_*T*_ = 0.5: *β*_ADHD-PGS_ (95% CI_Bootstrap_) = 0.019 (−0.013, 0.05), *p* = 0.26) or for a change in polygenic effect over time (*β*_ADHD-PGS × age_(95% CI_Bootstrap_) = 0.002 (−9 × 10^−5^, 0.0049, *p* = 0.10).

**Fig. 4:**
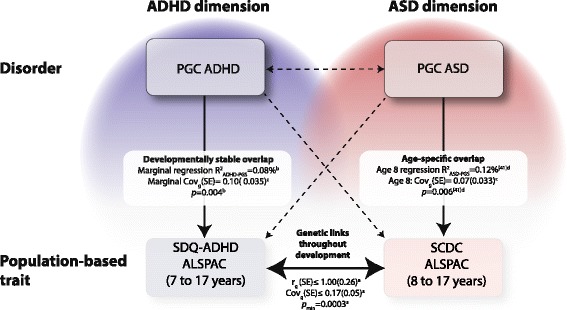
Genetic relationships between ASD and ADHD symptoms in clinical and population-based samples (as tagged by common variation). *Cross-disorder genetic overlap:* The Psychiatric Genomics Consortium (PGC) has previously reported [22] genetic correlations (*r*_g_) and covariances (Cov_g_) between PGC-ASD and PGC-ADHD (*r*_g_(SE) = −0.13 (0.09), Cov_g_(SE) = −0.026 (0.017), *p* = 0.13) using genetic-relationship-matrix restricted maximum likelihood (GREML) analyses that provided little evidence for cross-disorder genetic overlap. *Cross-trait genetic overlap:* Genetic correlations and covariances between standardised SCDC (at 8, 11, 14 and 17 years) and SDQ-ADHD scores (at 7, 10, 12, 13 and 17 years) in ALSPAC, as estimated using bivariate GREML (a), provided evidence for shared genetic links throughout childhood and adolescence (see the ‘Results’ section). *Uni-dimensional trait-disorder genetic overlap:* The association between SDQ-ADHD scores in ALSPAC and ADHD-PGS (polygenic risk scores (PGS)) was developmentally stable across development, as predicted by linear mixed models (b). A marginal estimate of regression *R* ^2^ and a marginal estimate of the expected genetic covariance, as estimated with the Avengeme software (c), are shown (see the ‘Results’ section). As previously reported [41], the association between ASD risk-increasing alleles and SCDC scores in ALSPAC was strongest at age 8 years. Regression *R* ^2^ estimates at age 8 years are shown, based on an age-specific analysis using standardised scores and linear regression models (d) [41]. The expected genetic covariance was estimated with Avengeme software (c) (Cov_g_(95% CI) = 0.072 (0.0082,0.14) (approximated SE = 0.033)). *Cross-dimensional trait-disorder genetic overlap:* There was little support for association between ASD-PGS and SDQ-ADHD or association between ADHD-PGS and SCDC scores in ALSPAC (see the ‘Results’ section, *p* > experiment-wise error rate *p* = 0.01). All polygenic scoring analyses are shown for a PGS threshold (*P*_*T*_) of 0.5. Genetic relationships reaching statistical significance are shown as *solid lines* and as *dashed lines* otherwise. Previous reports based on linkage disequilibrium score genetic correlations [23, 28] are omitted for clarity. *ADHD* attention deficit hyperactivity disorder, *ALSPAC* Avon Longitudinal Study of Parents and Children, *ASD* autism spectrum disorder, *PGC-ADHD* ADHD collection of the PGC, *PGC-ASD* ASD collection of the PGC, *SCDC* Social and Communication Disorders Checklist, *SDQ-ADHD* ADHD subscale of the Strength and Difficulties Questionnaire

A schematic summary of our results, in combination with previous findings reported by the PGC, is shown in Fig. 4.

## Discussion

Our findings provide strong evidence for shared genetic influences between population-based social-communication difficulties and ADHD symptoms during the course of child and adolescent development, as tagged by common genetic variants. Furthermore, population-based traits and disorders were genetically linked within the ASD and within the ADHD dimension, as suggested by this and previous analyses [41], although there was little support for cross-dimensional trait-disorder overlap, with respect to genetic risk for neither clinical ADHD nor clinical ASD.

Our study shows that in the general population, genetic influences between social-communication difficulties and ADHD symptoms, as tagged by common genetic markers, are shared across a ~10-year period spanning childhood and adolescence. These findings are in agreement with twin study findings in childhood [4, 5], adolescence [31] and adulthood [3, 6]. Genetic correlations across traits reached a similar strength and magnitude as those shared between repeated measures of the same trait, with up to 100% shared genetic influences during late childhood/early adolescence. At this age, genetic covariances contributed to more than a third of the observed cross-trait phenotypic correlation. Moreover, in absolute terms, the genetic contribution to phenotypic correlation across traits and between measures of the same trait was nearly identical. Thus, our study suggests that ASD and ADHD dimensions during late childhood and early adolescence, as measured by SCDC and SDQ scores, appear to have no clearly defined boundaries at the level of genetic variation tagged by common SNPs. This finding is consistent with a peak in ADHD and ASD symptom co-occurrence during adolescence [9]. In addition, our results on cross-trait overlap within (near) adult populations extend twin-based findings in adults and suggest that shared genetic links with ADHD symptoms may involve not only repetitive autistic symptoms [6] and social impairment [3] but also social-communication difficulties.

Joint genetic influences implicated in both ADHD symptoms and social-communication difficulties may include variation within K-RAS upregulated genes [53] explaining up to 3 and 6% of trait variation, respectively, especially during later adolescence. The human K-RAS gene is an isoform of the RAS oncogene-encoding GDP/GTP-binding proteins acting as intracellular signal transducers (OMIM 190070), and RAS proteins play a vital role in human tissue signalling, including proliferation and differentiation. K-RAS signalling upregulated genes involve ~200 loci [53], some of which are ASD candidate genes such as *RELN* [63] or are implicated in ASD- and ADHD-related metabotropic glutamate receptor networks based on copy number variation (CNV) analyses, such as *GRM3* [64, 65]. A follow-up study provided, however, little evidence that genetic variation within the entire network of metabotropic glutamate receptors [64, 65] (~266 loci) accounts for genetic variance in both traits (*p* = 0.25, data not shown). This may refer to potential differences between traits, disorders and different types of genetic markers. The variance contributions explained by K-RAS signalling upregulated genes increased for both, SDQ-ADHD and SCDC scores, during development and were largest during later adolescence with age-dependent developmental but no trait-dependent changes. Thus, our results do not necessarily imply that developmentally shared genetic influences between ADHD symptoms and social-communication difficulties are genetically stable but that aetiological mechanisms in both traits are developmentally coupled. This clearly underlines the need to investigate genetic trait variances jointly as part of multivariate analysis approaches, once these methods become computationally feasible.

Besides developmental continuity in genetic overlap, we also noted a drop in cross-trait genetic relationships involving ADHD symptoms at age 12, irrespective of their genetic variance and their genetic links with ADHD symptoms earlier and later during development. This change in cross-trait genetic correlations was not captured by cross-trait phenotypic correlations, suggesting that phenotypic links may not always represent an accurate approximation of the underlying genetic architecture. It has been shown that children with high ADHD symptoms at age 12 may follow very different developmental paths [66] including trajectories of persistent, childhood limited or intermediate ADHD problems that become apparent during the following stages of development. It is thus possible to hypothesise that ADHD symptoms measured at age 12 years are genetically more heterogeneous than scores assessed at younger or older ages, and this may have relevance for researchers conducting genome-wide analyses of ADHD behaviour in large samples. Thus, multivariate decompositions of phenotypic variance are required, which are computationally not yet feasible, to disentangle the underlying variance components shared between ADHD symptoms before and after this age. In addition, we cannot exclude the presence of gender-specific effects, although the power [67] to investigate such effects was too low in our study (the power is 0.16, assuming 2000 same-sex individuals and a heritability of 0.18 as observed for SDQ-ADHD scores at age 13).

Uni-dimensional analyses of trait-disorder overlap, conducted within this and previous studies [41], identified a developmentally stable genetic overlap between population-based ADHD symptoms of the combined hyperactive-impulsive/inattentive type and clinical ADHD, as well as an age-specific overlap between social-communication difficulties during childhood and clinical ASD [28, 41]. These findings advocate that the investigated population-based traits each represent dimensional phenotypes mapping to an underlying ADHD and ASD continuum respectively.

However, investigating cross-dimensional links between traits and disorder, we found neither robust evidence for shared genetic aetiologies between social-communication difficulties and clinical ADHD nor between ADHD symptoms and clinical ASD. These findings are consistent with negligible genetic correlations between clinical ASD and clinical ADHD in the PGC samples [22, 23]. Using PGC-ASD as a discovery sample and ALSPAC as target (for example SDQ-ADHD scores at age 12), the power [58] to detect cross-dimensional trait-disorder overlap is low (0.23), assuming a type I error rate of 0.05 and a cross-dimensional trait-disorder genetic covariance that corresponds to about half of the uni-dimensional one (see Fig. 4). Similar power estimates (0.21) were also obtained when using PGC-ADHD as a discovery sample and ALSPAC as target (for example SCDC scores at age 8). Lack of evidence for cross-dimensional trait-disorder overlap is thus partly a consequence of small clinical discovery samples [59], suggesting that much larger clinical sample sizes are required to reliably detect cross-dimensional trait-disorder relationships.

The fraction of phenotypic trait variance that can be accounted for by risk-increasing alleles for disorder is, nonetheless, small, even for population-based symptoms that have been mapped to the same behavioural dimension (<1%). In light of aetiological differences between subclinical variation in population-based symptoms and severe neurodevelopmental conditions, it is thus likely that a considerable proportion of genetic factors contributing to shared genetic links between social-communication difficulties and ADHD symptoms on the general population level will be non-specific to either disorder. It is furthermore conceivable that genetic links between comorbid ADHD and ASD symptoms are domain dependent. For example, inattentive symptoms tend to be more persistent than hyperactive-impulsive problems, as the latter tend to resolve with progressing age [68], possibly pointing to distinct genetic underpinnings [31]. Thus, investigations of samples with longitudinal information on behavioural subdomains may support analyses of comorbid ADHD and ASD symptoms.

A limitation of our study is that we cannot fully exclude the possibility of transformation-related bias with respect to the studied population-based traits. However, genetic links between ADHD symptoms and clinical ADHD and between SCDC scores and clinical ASD, as previously reported [41], were confirmed using untransformed data. In addition, we cannot exclude the possibility that phenotypic relationships between population-based traits are upward-biased due to enhanced variance sharing because of mother-report, although this is unlikely to affect the reported genetic relationships in children. Furthermore, participants with behavioural problems are more likely to discontinue participation in longitudinal studies [69, 70]. Thus, participants with higher scores on the SCDC and/or the SDQ-ADHD subscale are more likely to drop out compared to participants with lower scores, as both instruments are known to capture also behavioural difficulties [37, 40]. Longitudinal analyses of trait-disorder overlap accounting for unequal sample dropout through bootstrapping identified, however, little evidence for bias. Note that there is also little evidence for sex-specific attrition in ALSPAC [69]. Finally, our findings of stability and change in cross-trait genetic relationships during development are representative of an entire cohort. Thus, our results do not allow inferences on participants with extreme behavioural scores during development, who represent only a small proportion of the ALSPAC children studied (≤10%) [66].

## Conclusions

In the general population, genetic aetiologies between social-communication difficulties and ADHD symptoms are linked throughout childhood and adolescence and may implicate similar biological pathways that are developmentally coupled. Risk-increasing alleles for disorder can account, however, only for a very small proportion of trait variance, even for symptoms mapping to the same behavioural dimension, suggesting that much larger clinical samples are required to reliably detect cross-dimensional trait-disorder relationships.

## Additional files

Additional file 1: Additional note. Selection of SDQ-ADHD measures. Additional note. Meta-analysis of correlated test statistics from pathway analysis. Additional note. Additional references. Additional note. Web resources. **Table S1.** Descriptives of SDQ-ADHD and SCDC scores in ALSPAC. **Table S2.** Phenotypic correlations of SDQ-ADHD scores in ALSPAC. **Table S3.** Phenotypic correlations of SCDC scores in ALSPAC. **Table S4.** Univariate GREML of SDQ-ADHD scores in ALSPAC. **Table S5.** Univariate GREML of SCDC scores in ALSPAC. **Table S6.** Bivariate GREML of SDQ-ADHD scores in ALSPAC. **Table S7.** Bivariate GREML of SCDC scores in ALSPAC. **Table S8.** Bivariate GREML and Pearson correlations of SDQ-ADHD and SCDC scores in ALSPAC. **Table S10.** Association between ADHD polygenic scores and SDQ-ADHD scores in ALSPAC. **Table S11.** Association between ASD polygenic scores and SDQ-ADHD scores in ALSPAC. **Table S12.** Association between ADHD polygenic scores and SCDC scores in ALSPAC. (DOCX 92 kb)

Additional file 2: Table S9. Pathway-based dissection of additive genetic variance in SDQ-ADHD and SCDC scores according to 50 molecular signatures database hallmark gene set collections. (XLSX 20 kb)

## Abbreviations

ADHD: Attention-deficit/hyperactivity disorder
ALSPAC: Avon Longitudinal Study of Parents and their Children
ASD: Autism spectrum disorder
CNV: Copy number variation
DSM: Diagnostic and Statistical Manual of Mental Disorders
GCTA: Genome-wide Complex Trait Analysis
GREML: Genetic-relationship-matrix restricted maximum likelihood
GRM: Genetic-relationship-matrix
GRM3: Metabotropic glutamate receptor 3
GWAS: Genome-wide association study
h^2^: Heritability
HapMap3: Third phase of the International HapMap project
HapMap3 CEU: Utah residents with Northern and Western European ancestry from the HapMap 3 project
HapMap3 TSI: Population sample from Toscani in Italia from the HapMap 3 project
HWE: Hardy-Weinberg equilibrium
ICD: International Classification of Diseases
K-RAS: Isoform of the RAS oncogene-encoding GDP/GTP-binding proteins
MAF: Minor allele frequency
MSigDB: Molecular signatures database
OLS: Ordinary least square
OMIM: Online Mendelian Inheritance in Man
PGC: Psychiatric Genomics Consortium
RAS: Rat sarcoma proto-onkogen
*r*_e_: Residual correlation
RELN: Reelin
*r*_g_: Genetic correlation
*r*_p_: Phenotypic correlation
SCDC: Social Communication Disorder Checklist
SDQ: Strengths and Difficulties Questionnaire
SDQ-ADHD: ADHD subscale of the Strengths and Difficulties Questionnaire
SE: Standard error
SNP: Single-nucleotide polymorphism
