# Supplementary material for: Shared genetic influences between dimensional ASD and ADHD symptoms during child and adolescent development
Source: Mol Autism. 2017 Apr 4;8:18. doi: 10.1186/s13229-017-0131-2 (PMC5379648; doi:10.1186/s13229-017-0131-2)
Supplement: Supplementary file 1 — Additional note. Selection of SDQ-ADHD measures. Additional note. Meta-analysis of correlated test statistics from pathway analysis. Additional note. Additional references. Additional note. Web resources. Table S1. Descriptives of SDQ-ADHD and SCDC scores in ALSPAC. Table S2. Phenotypic correlations of SDQ-ADHD scores in ALSPAC. Table S3. Phenotypic correlations of SCDC scores in ALSPAC. Table S4. Univariate GREML of SDQ-ADHD scores in ALSPAC. Table S5. Univariate GREML of SCDC scores in ALSPAC. Table S6. Bivariate GREML of SDQ-ADHD scores in ALSPAC. Table S7. Bivariate GREML of SCDC scores in ALSPAC. Table S8. Bivariate GREML and Pearson correlations of SDQ-ADHD and SCDC scores in ALSPAC. Table S10. Association between ADHD polygenic scores and SDQ-ADHD scores in ALSPAC. Table S11. Association between ASD polygenic scores and SDQ-ADHD scores in ALSPAC. Table S12. Association between ADHD polygenic scores and SCDC scores in ALSPAC. (DOCX 92 kb) [file 13229_2017_131_MOESM1_ESM.docx]

**Additional material**

**Shared genetic influences between dimensional ASD and ADHD symptoms during child and adolescent development**

**Additional notes**

- Selection of SDQ-ADHD measures
- Meta-analysis of correlated test statistics from pathway analysis
- Additional references
- Web resources

**Additional tables**

- Table S1: Descriptives of SDQ-ADHD and SCDC scores in ALSPAC
- Table S2: Phenotypic correlations of SDQ-ADHD scores in ALSPAC
- Table S3: Phenotypic correlations of SCDC scores in ALSPAC
- Table S4: Univariate GREML of SDQ-ADHD scores in ALSPAC
- Table S5: Univariate GREML of SCDC scores in ALSPAC
- Table S6: Bivariate GREML of SDQ-ADHD scores in ALSPAC
- Table S7: Bivariate GREML of SCDC scores in ALSPAC
- Table S8: Bivariate GREML and Pearson correlations of SDQ-ADHD and SCDC scores in ALSPAC
- Table S9: Pathway-based dissection of additive genetic variance in SDQ-ADHD and SCDC scores according to 50 molecular signatures database hallmark gene set collections (file A9.xlsx)
- Table S10: Association between ADHD polygenic scores and SDQ-ADHD scores in ALSPAC
- Table S11: Association between ASD polygenic scores and SDQ-ADHD scores in ALSPAC
- Table S12: Association between ADHD polygenic scores and SCDC scores in ALSPAC

**Supplementary methods**

Selection of SDQ-ADHD measures

Mother-reports on their children’s hyperactive-impulsive and inattentive behaviour were obtained at 7, 10, 12, 13 and 17 years of age. An additional SDQ measure at 8 years of age was excluded due to potential bias. The administration of the questionnaire coincided with the recruitment of additional children into the study, resulting in late questionnaire returns (Jean Golding, personal communication). The reported age range for children using this questionnaire was considerably wider (7.83 to 14 years) and had approximately twice the variance (σ^2^=0.25) than the observed age ranges and variances for children at slightly younger ages (7 year assessment: range 6.67 to 8.2 years; σ^2^=0.11) and at slightly older ages (10 year assessment: range 9.5 years to 10.75 years; σ^2^=0.12) (Table S1).

Meta-analysis of correlated test statistics from pathway analysis

Genetic variances of SCDC and SDQ-ADHD scores (four SCDC scores at 8, 11, 14 and 17 years and five SDQ-ADHD scores at 7, 10 12, 13 and 17 years) were combined using an inverse variance weighted meta-analysis statistic, assuming multivariate normality [1],

$S=\frac{e^{T}{(RW)}^{-1}B{(e^{T}{(RW)}^{-1}B)}^{T}}{e^{T}{(WRW)}^{-1}e}$ (1)

which follows a χ^2^ distribution with one degree of freedom. B is a vector of test statistics (B_1_,...,B_K_), where each test statistic is defined as the ratio of the additive genetic variance estimate explained by a selected pathway for measures K and its SE. R is approximated by the K x K phenotypic correlation matrix between all traits, i.e. rank-transformed SDQ-ADHD and SCDC scores. e^T^= (1,...,1) has length K and W is a diagonal matrix of weights for the individual test statistics (inverse variance).

**Additional references**

1. Zhu X, Feng T, Tayo BO, Liang J, Young JH, Franceschini N, et al. Meta-analysis of Correlated Traits via Summary Statistics from GWASs with an Application in Hypertension. Am. J. Hum. Genet. 2015;96:21–36.

2. St Pourcain B, Skuse DH, Mandy WP, Wang K, Hakonarson H, Timpson NJ, et al. Variability in the common genetic architecture of social-communication spectrum phenotypes during childhood and adolescence. Mol. Autism. 2014;5:18.

**Web resources**

ALSPAC: <http://www.bris.ac.uk/alspac/>

PGC: <http://www.med.unc.edu/pgc/>

**Supplementary tables**

**Table S1:** Descriptives of SDQ-ADHD and SCDC scores in ALSPAC

| Trait | Age(years)[range] | Male/Female | Mean(SD) [range] | Kurtosis | Skewness | N |
| --- | --- | --- | --- | --- | --- | --- |
| SDQ-ADHD | | | | | | |
| 7 | 6.8(0.11)[6. 7;8.2] | 2864/2748 | 3(2.35)[0;10] | 2.85 | 0.62 | 5612 |
| 10 | 9.7(0.12)[9.5;10.8] | 2868/2810 | 3(2.24)[0;10] | 3.31 | 0.83 | 5678 |
| 12 | 11.7(0.13)[11.6;13.8] | 2613/2646 | 2(2.22)[0;10] | 3.61 | 0.93 | 5259 |
| 13 | 13.2(0.18)[12.9;16.1] | 2524/2548 | 3(2.22)[0;10] | 3.25 | 0.78 | 5072 |
| 17 | 16.8(0.36)[16.5;18.3] | 2019/2145 | 2(2.12)[0;10] | 2.85 | 0.62 | 4164 |
| SCDC | |  |  |  |  |  |
| 8 | 7.7(0.14)[7.5;9.3] | 2842/2709 | 2(3.71)[0;24] | 9.12 | 2.19 | 5551 |
| 11 | 10.7(0.13)[10.5;13.8] | 2751/2709 | 1(3.51)[0;24] | 10.53 | 2.46 | 5460 |
| 14 | 13.9(0.15)[13. 7;16.1] | 2529/2531 | 1(3.59)[0;24] | 9.08 | 2.20 | 5060 |
| 17 | 16.8(0.36)[16.5;18.3] | 2024/2150 | 1(3.79)[0;24] | 7.09 | 1.89 | 4174 |

ALSPAC - Avon Longitudinal Study of Parents and Children; SCDC - Social and Communication Disorders Checklist; SDQ-ADHD - ADHD-subscale of the Strength and Difficulties Questionnaire; The kurtosis for the standard normal distribution is 3 and the skewness is 0

**Table S2:** Phenotypic correlations of SDQ-ADHD scores in ALSPAC

| **Age in years** | | | | | |
| --- | --- | --- | --- | --- | --- |
|  | **7** | **10** | **12** | **13** | **17** |
| **7** | 1.00 | 0.65 | 0.60 | 0.56 | 0.46 |
| **10** | 0.66 | 1.00 | 0.67 | 0.63 | 0.52 |
| **12** | 0.61 | 0.68 | 1.00 | 0.69 | 0.55 |
| **13** | 0.57 | 0.63 | 0.70 | 1.00 | 0.59 |
| **17** | 0.46 | 0.52 | 0.56 | 0.59 | 1.00 |

ALSPAC - Avon Longitudinal study of Parents and Children; SDQ-ADHD - ADHD-subscale of the Strength and Difficulties Questionnaire

Lower triangle: Spearman’s rank correlation using pairwise complete observations; Upper triangle: Pearson product moment correlation using rank-transformed scores adjusted for age, sex and the two most significant principal components

**Table S3:** Phenotypic correlations of SCDC scores in ALSPAC

| **Age in years** | | | | |
| --- | --- | --- | --- | --- |
|  | **8** | **11** | **14** | **17** |
| **8** | 1.00 | 0.61 | 0.50 | 0.38 |
| **11** | 0.57 | 1.00 | 0.56 | 0.41 |
| **14** | 0.49 | 0.57 | 1.00 | 0.51 |
| **17** | 0.39 | 0.45 | 0.56 | 1.00 |

ALSPAC - Avon Longitudinal study of Parents and Children; SCDC - Social and Communication Disorders Checklist

Lower triangle: Spearman’s rank correlation using pairwise complete observations; Upper triangle: Pearson product moment correlation using rank-transformed scores adjusted for age, sex and the two most significant ancestry-informative principal components; Estimates correspond closely to previously published correlations[2]

**Table S4:** Univariate GREML of SDQ-ADHD scores in ALSPAC

| **Age in years** | | | | | | |  | |  | |  | |  |
| --- | --- | --- | --- | --- | --- | --- | --- | --- | --- | --- | --- | --- | --- |
|  | **Rank-transformed** | | | | | | **Untransformed scores** | | | | | |  |
|  | **h^2^(SE)** | **Var_g_** | ***p*** | | **N** | | **h^2^(SE)** | | ***p*** | | **N** | |  |
| **7** | 0.10(0.07) | 0.10(0.07) | | 0.070 | | 5185 | | 0.09(0.07) | | 0.095 | | 5185 | |
| **10** | 0.14(0.07) | 0.14(0.07) | | 0.020 | | 5235 | | 0.14(0.07) | | 0.017 | | 5235 | |
| **12** | 0.19(0.07) | 0.19(0.07) | | 0.0020^a^ | | 4886 | | 0.15(0.07) | | 0.010 | | 4886 | |
| **13** | 0.18(0.07) | 0.18(0.07) | | 0.0030^a^ | | 4735 | | 0.16(0.07) | | 0.010 | | 4735 | |
| **17** | 0.15(0.09) | 0.15(0.09) | | 0.04 | | 3968 | | 0.12(0.09) | | 0.079 | | 3968 | |

ALSPAC - Avon Longitudinal study of Parents and Children; GREML - Genetic-relationship-matrix restricted maximum likelihood; h^2^ - heritability; SDQ-ADHD - ADHD-subscale of the Strength and Difficulties Questionnaire; Rank-transformed - Rank-transformed scores; Untransformed - Untransformed scores (sensitivity analysis only); Var_g_ - Genetic variance; a - *p*<0.05 after adjustment for multiple testing

All GREML-h^2^ estimates were adjusted for age and sex and the two most significant ancestry-informative principal components. Differences compared with the total sample N are due to the exclusion of individuals with a relatedness of ≥ 2.5%. Note that for rank-transformed traits, estimates of SNP-h^2^ are equivalent to estimates of Var_g_, as the phenotypic variance has been standardised to one

**Table S5:** Univariate GREML of SCDC scores in ALSPAC

| **Age in years** | | | | | | |  | |  | |  | |  |
| --- | --- | --- | --- | --- | --- | --- | --- | --- | --- | --- | --- | --- | --- |
|  | **Rank-transformed** | | | | | | **Untransformed scores^b^** | | | | | |  |
|  | **h^2^(SE)** | **Var_g_** | ***p*** | | **N** | | **h^2^(SE)** | | ***p^a^*** | | **N** | |  |
| **8** | 0.24(0.07) | 0.24(0.07) | | 7.0x10^-5 a^ | | 5136 | | 0.11(0.07) | | 0.043 | | 5136 | |
| **11** | 0.16(0.07) | 0.16(0.07) | | 0.0050 ^a^ | | 5056 | | 0.04(0.07) | | 0.28 | | 5056 | |
| **14** | 0.08(0.07) | 0.08(0.07) | | 0.10 | | 4735 | | 0.07(0.07) | | 0.15 | | 4735 | |
| **17** | 0.45(0.08) | 0.45(0.09) | | 3.0x10^-9 a^ | | 3977 | | 0.22(0.09) | | 0.0051^a^ | | 3977 | |

ALSPAC - Avon Longitudinal study of Parents and Children; GREML - Genetic-relationship-matrix restricted maximum likelihood; h^2^ - heritability; SCDC - Social and Communication Disorders Checklist; Rank-transformed - Rank-transformed scores; Untransformed - Untransformed scores (sensitivity analysis only); Var_g_ - Genetic variance; a - *p*<0.05 after adjustment for multiple testing

All GREML-h^2^ estimates were adjusted for age and sex and the two most significant ancestry-informative principal components. Differences compared with the total sample N are due to the exclusion of individuals with a relatedness of ≥ 2.5%. Note that for rank-transformed traits, estimates of SNP-h^2^ are equivalent to estimates of Var_g_, as the phenotypic variance has been standardised to one. Findings correspond closely to previously published estimates[2]

**Table S6:** Bivariate GREML of SDQ-ADHD scores in ALSPAC

| **r_g_ (SE), *p* (lower triangle) and Cov_g_ (upper triangle)** | | | | | |
| --- | --- | --- | --- | --- | --- |
| **Age** | **7** | **10** | **12** | **13** | **17** |
| **7** | - | 0.13(0.05) | 0.09(0.05) | 0.10(0.05) | 0.07(0.06) |
| **10** | 0.86(0.17), *p*=8x10^-3 a^ | - | 0.17(0.06) | 0.13(0.06) | 0.08(0.06) |
| **12** | 0.65(0.21),  *p*=0.04 | 0.94(0.13),  *p*=7x10^-4 a^ | - | 0.15(0.06) | 0.03(0.06) |
| **13** | 0.73(0.25),  *p*=0.03 | 0.76(0.17),  *p*=9x10^-3 a^ | 0.76(0.13),  *p*=4x10^-3 a^ | - | 0.13(0.06) |
| **17** | 0.66(0.43),  *p*=0.10 | 0.52(0.25),  *p*=0.07 | 0.20(0.31),  *p*=0.30 | 0.88(0.24),  *p*=0.01 ^a^ | - |

ALSPAC - Avon Longitudinal study of Parents and Children; Cov_g_ - Genetic covariance; GREML - Genetic-relationship-matrix restricted maximum likelihood; r_g_ - genetic correlation; SDQ-ADHD - ADHD-subscale of the Strength and Difficulties Questionnaire (rank-transformed); a - *p*<0.05 after adjustment for multiple testing

**Table S7:** Bivariate GREML of SCDC scores in ALSPAC

| **r_g_ (SE), *p* (lower triangle) and Cov_g_ (upper triangle)** | | | | |
| --- | --- | --- | --- | --- |
| **Age** | **8** | **11** | **14** | **17** |
| **8** | - | 0.18(0.05) | 0.10(0.05) | 0.17(0.06) |
| **11** | 0.93(0.14),  *p*=1x10^-4 a^ | - | 0.11(0.05) | 0.11(0.06) |
| **14** | 0.78(0.31),  *p*=0.02 | 0.82(0.24),  *p*=0.02 | - | 0.19(0.06) |
| **17** | 0.50(0.14),  *p*=9x10^-4 a^ | 0.38(0.16),  *p*=0.02 | 0.95(0.34),  *p*=2x10^-7 a^ | - |

ALSPAC - Avon Longitudinal study of Parents and Children; Cov_g_ - Genetic covariance; GREML - Genetic-relationship-matrix restricted maximum likelihood; GREML-r_g_ - Genetic correlation; SCDC - Social and Communication Disorders Checklist (rank-transformed); a - *p*<0.05 after adjustment for multiple testing; r_g_(SE), *p* are shown in the lower triangle and Cov_g_ in the upper triangle

**Table S8:** Bivariate GREML and Pearson correlations of SDQ-ADHD and SCDC scores in ALSPAC

| **r_g_ (SE), *p^a^*** | | | | |  |
| --- | --- | --- | --- | --- | --- |
| **Age** | **SCDC** | **8** | **11** | **14** | **17** |
| **SDQ** |  |  |  |  |  |
| **7** |  | 0.80(0.25),  *p*=6x10^-3 a^ | 1.00(0.35),  *p*=2x10^-3 a^ | 1.00(0.79),  *p*=0.50 | 0.52(0.29),  *p*=0.03 |
| **10** |  | 0.62(0.20),  *p*=0.01 ^a^ | 0.74(0.23),  *p*=8x10^-3 a^ | 0.89(0.40),  *p*=0.02 | 0.53(0.19),  *p*=5x10^-3 a^ |
| **12** |  | 0.39(0.19),  *p*=0.05 | 0.44(0.21),  *p*=0.05 | 0.48(0.30),  *p*=0.10 | 0.13(0.19),  *p*=0.30 |
| **13** |  | 0.72(0.19),  *p*=2x10^-3 a^ | 1.00(0.26),  *p*=3x10^-4 a^ | 0.80(0.34),  *p*=0.03 | 0.42(0.17),  *p*=0.02 |
| **17** |  | 0.73(0.37),  *p*=0.02 | 0.92(0.43),  *p*=0.01 ^a^ | 1.00(0.43),  *p*=0.01 ^a^ | 0.80(0.26),  *p*=3x10^-3 a^ |
| **Cov_g_(SE)** |  |  |  |  |  |
| **Age** | **SCDC** | **8** | **11** | **14** | **17** |
| **SDQ-ADHD** |  |  |  |  |  |
| **7** |  | 0.13(0.05) | 0.14(0.05) | 0.07(0.05) | 0.10(0.06) |
| **10** |  | 0.11(0.05) | 0.12(0.05) | 0.10(0.05) | 0.14(0.06) |
| **12** |  | 0.09(0.05) | 0.08(0.05) | 0.07(0.05) | 0.04(0.06) |
| **13** |  | 0.15(0.05) | 0.17(0.05) | 0.10(0.05) | 0.12(0.06) |
| **17** |  | 0.11(0.06) | 0.12(0.06) | 0.12(0.06) | 0.18(0.07) |
| **r_p_(SE)** |  |  |  |  |  |
| **Age** | **SCDC** | **8** | **11** | **14** | **17** |
| **SDQ-ADHD** |  |  |  |  |  |
| **7** |  | 0.43 | 0.36 | 0.3 | 0.27 |
| **10** |  | 0.44 | 0.41 | 0.37 | 0.33 |
| **12** |  | 0.39 | 0.40 | 0.37 | 0.32 |
| **13** |  | 0.40 | 0.41 | 0.42 | 0.35 |
| **17** |  | 0.34 | 0.36 | 0.39 | 0.50 |

Age - Age in years; ALSPAC - Avon Longitudinal study of Parents and Children; Cov_g_ - Genetic covariance; GREML - Genetic-relationship-matrix restricted maximum likelihood; r_g_ - Genetic correlation; r_P_ - Pearson product moment correlation based on rank-transformed traits; SCDC - Social and Communication Disorders Checklist(rank-transformed); SDQ-ADHD - ADHD-subscale of the Strength and Difficulties Questionnaire (rank-transformed); a - *p*<0.05 after adjustment for multiple testing

**Table S9:** Pathway-based dissection of additive genetic variance in SDQ-ADHD and SCDC scores according to 50 molecular signatures database hallmark gene set collections (file A9.xlsx)

**Table S10:** Association between ADHD polygenic scores and SDQ-ADHD scores in ALSPAC

| **SDQ-ADHD** | **PGS bin** | **Beta (SE)** | ***p*** | **Adj-R^2^ (%)** |
| --- | --- | --- | --- | --- |
| 7 y (N=5612) | P_T_<0.001 | 0.002(0.01) | 0.9 | na |
|  | P_T_<0.01 | 0.009(0.01) | 0.52 | na |
|  | P_T_<0.05 | 0.014(0.01) | 0.3 | na |
|  | P_T_<0.1 | 0.022(0.01) | 0.11 | na |
|  | P_T_<0.3 | 0.035(0.01) | 0.009 | 0.1 |
|  | P_T_<0.5 | 0.033(0.01) | 0.013 | 0.09 |
|  | P_T_<0.7 | 0.032(0.01) | 0.015 | 0.09 |
|  | P_T_<0.9 | 0.032(0.01) | 0.018 | 0.08 |
|  | P_T_<1 | 0.032(0.01) | 0.018 | 0.08 |
| 10 y(N=5678) | P_T_<0.001 | 0.018(0.01) | 0.18 | na |
|  | P_T_<0.01 | 0.019(0.01) | 0.16 | na |
|  | P_T_<0.05 | 0.012(0.01) | 0.38 | na |
|  | P_T_<0.1 | 0.02(0.01) | 0.14 | na |
|  | P_T_<0.3 | 0.022(0.01) | 0.093 | na |
|  | P_T_<0.5 | 0.027(0.01) | 0.042 | 0.05 |
|  | P_T_<0.7 | 0.024(0.01) | 0.067 | na |
|  | P_T_<0.9 | 0.024(0.01) | 0.072 | na |
|  | P_T_<1 | 0.024(0.01) | 0.07 | na |
| 12 y (N=5259) | P_T_<0.001 | 0.005(0.01) | 0.73 | na |
|  | P_T_<0.01 | 0.01(0.01) | 0.48 | na |
|  | P_T_<0.05 | 0.016(0.01) | 0.26 | na |
|  | P_T_<0.1 | 0.021(0.01) | 0.12 | na |
|  | P_T_<0.3 | 0.021(0.01) | 0.14 | na |
|  | P_T_<0.5 | 0.024(0.01) | 0.084 | na |
|  | P_T_<0.7 | 0.024(0.01) | 0.082 | na |
|  | P_T_<0.9 | 0.024(0.01) | 0.079 | na |
|  | P_T_<1 | 0.024(0.01) | 0.079 | na |
| 13 y (N=5072) | P_T_<0.001 | 0.011(0.01) | 0.45 | na |
|  | P_T_<0.01 | 0.024(0.01) | 0.085 | na |
|  | P_T_<0.05 | 0.028(0.01) | 0.046 | 0.06 |
|  | P_T_<0.1 | 0.035(0.01) | 0.014 | 0.1 |
|  | P_T_<0.3 | 0.031(0.01) | 0.031 | 0.07 |
|  | P_T_<0.5 | 0.033(0.01) | 0.017 | 0.09 |
|  | P_T_<0.7 | 0.033(0.01) | 0.02 | 0.09 |
|  | P_T_<0.9 | 0.033(0.01) | 0.02 | 0.09 |
|  | P_T_<1 | 0.033(0.01) | 0.019 | 0.09 |
| 17 y (N=4164) | P_T_<0.001 | -0.009(0.02) | 0.55 | na |
|  | P_T_<0.01 | 0.034(0.02) | 0.032 | 0.09 |
|  | P_T_<0.05 | 0.03(0.02) | 0.054 | na |
|  | P_T_<0.1 | 0.043(0.02) | 0.0058 | 0.16 |
|  | P_T_<0.3 | 0.037(0.02) | 0.018 | 0.11 |
|  | P_T_<0.5 | 0.042(0.02) | 0.0073 | 0.15 |
|  | P_T_<0.7 | 0.039(0.02) | 0.013 | 0.12 |
|  | P_T_<0.9 | 0.039(0.02) | 0.013 | 0.13 |
|  | P_T_<1 | 0.039(0.02) | 0.012 | 0.13 |

Polygenic scores for risk-increasing alleles (PGS) in the PGC-ADHD sample were constructed in ALSPAC and then Z-standardised. Rank-transformed SDQ-ADHD scores were regressed on ADHD-PGS using linear regression, and are shown by PGS threshold (P_T_); Adjusted regression R^2^ (Adj-R^2^) is reported for signals with *p*<0.05; ALSPAC - Avon Longitudinal Study of Parents and Children; ADHD - Attention Deficit Hyperactivity Disorder; na - Not available; PGC - Psychiatric Genomics Consortium ; SDQ-ADHD - ADHD-subscale of the Strength and Difficulties Questionnaire (rank-transformed); y - years

**Table S11:** Association between ASD polygenic scores and SDQ-ADHD scores in ALSPAC

| **SDQ-ADHD** | **PGS bin** | **Beta (SE)** | ***p*** | **Adj-R^2^ (%)** |
| --- | --- | --- | --- | --- |
| 7 y (N=5612) | P_T_<0.001 | 0.017(0.01) | 0.2 | na |
|  | P_T_<0.01 | 0.014(0.01) | 0.3 | na |
|  | P_T_<0.05 | 0.008(0.01) | 0.57 | na |
|  | P_T_<0.1 | 0.007(0.01) | 0.62 | na |
|  | P_T_<0.3 | 0.011(0.01) | 0.41 | na |
|  | P_T_<0.5 | 0.01(0.01) | 0.46 | na |
|  | P_T_<0.7 | 0.009(0.01) | 0.52 | na |
|  | P_T_<0.9 | 0.008(0.01) | 0.54 | na |
|  | P_T_<1 | 0.008(0.01) | 0.54 | na |
| 10 y(N=5678) | P_T_<0.001 | 0.017(0.01) | 0.2 | na |
|  | P_T_<0.01 | -0.004(0.01) | 0.78 | na |
|  | P_T_<0.05 | -0.005(0.01) | 0.72 | na |
|  | P_T_<0.1 | -0.01(0.01) | 0.45 | na |
|  | P_T_<0.3 | -0.007(0.01) | 0.61 | na |
|  | P_T_<0.5 | -0.005(0.01) | 0.71 | na |
|  | P_T_<0.7 | -0.006(0.01) | 0.63 | na |
|  | P_T_<0.9 | -0.006(0.01) | 0.63 | na |
|  | P_T_<1 | -0.006(0.01) | 0.63 | na |
| 12 y (N=5259) | P_T_<0.001 | 0.023(0.01) | 0.089 | na |
|  | P_T_<0.01 | -0.001(0.01) | 0.96 | na |
|  | P_T_<0.05 | -0.006(0.01) | 0.67 | na |
|  | P_T_<0.1 | -0.011(0.01) | 0.42 | na |
|  | P_T_<0.3 | -0.006(0.01) | 0.66 | na |
|  | P_T_<0.5 | -0.003(0.01) | 0.81 | na |
|  | P_T_<0.7 | -0.004(0.01) | 0.77 | na |
|  | P_T_<0.9 | -0.004(0.01) | 0.77 | na |
|  | P_T_<1 | -0.004(0.01) | 0.77 | na |
| 13 y (N=5072) | P_T_<0.001 | 0.029(0.01) | 0.035 | 0.07 |
|  | P_T_<0.01 | 0(0.01) | 0.98 | na |
|  | P_T_<0.05 | 0.004(0.01) | 0.79 | na |
|  | P_T_<0.1 | -0.001(0.01) | 0.94 | na |
|  | P_T_<0.3 | 0(0.01) | 0.98 | na |
|  | P_T_<0.5 | 0.006(0.01) | 0.65 | na |
|  | P_T_<0.7 | 0.006(0.01) | 0.7 | na |
|  | P_T_<0.9 | 0.006(0.01) | 0.67 | na |
|  | P_T_<1 | 0.006(0.01) | 0.67 | na |
| 17 y (N=4164) | P_T_<0.001 | 0.006(0.02) | 0.68 | na |
|  | P_T_<0.01 | 0.001(0.02) | 0.93 | na |
|  | P_T_<0.05 | 0.002(0.02) | 0.91 | na |
|  | P_T_<0.1 | -0.006(0.02) | 0.7 | na |
|  | P_T_<0.3 | -0.007(0.02) | 0.63 | na |
|  | P_T_<0.5 | -0.006(0.02) | 0.7 | na |
|  | P_T_<0.7 | -0.006(0.02) | 0.7 | na |
|  | P_T_<0.9 | -0.006(0.02) | 0.71 | na |
|  | P_T_<1 | -0.006(0.02) | 0.72 | na |

Polygenic scores for risk-increasing alleles (PGS) in the PGC-ASD sample were constructed in ALSPAC and then Z-standardised. Rank-transformed SDQ-ADHD scores were regressed on ASD-PGS using linear regression, and are shown by PGS threshold (P_T_); Adjusted regression R^2^ (Adj-R^2^) is reported for signals with *p*<0.05; ALSPAC - Avon Longitudinal Study of Parents and Children; ASD - Autism Spectrum Disorders; na - Not available; PGC - Psychiatric Genomics Consortium ; SDQ-ADHD - ADHD-subscale of the Strength and Difficulties Questionnaire (rank-transformed); y - years

**Table S12:** Association between ADHD polygenic scores and SCDC scores in ALSPAC

| **SCDC** | **PGS bin** | **Beta (SE)** | ***p*** | **Adj-R^2^ (%)** |
| --- | --- | --- | --- | --- |
| 8 y (N=5551) | P_T_<0.001 | -0.001(0.01) | 0.94 | na |
|  | P_T_<0.01 | 0.004(0.01) | 0.77 | na |
|  | P_T_<0.05 | 0.001(0.01) | 0.95 | na |
|  | P_T_<0.1 | -0.002(0.01) | 0.91 | na |
|  | P_T_<0.3 | 0.002(0.01) | 0.87 | na |
|  | P_T_<0.5 | 0.006(0.01) | 0.68 | na |
|  | P_T_<0.7 | 0.006(0.01) | 0.65 | na |
|  | P_T_<0.9 | 0.005(0.01) | 0.69 | na |
|  | P_T_<1 | 0.006(0.01) | 0.68 | na |
| 11 y (N=5460) | P_T_<0.001 | -0.014(0.01) | 0.28 | na |
|  | P_T_<0.01 | -0.002(0.01) | 0.89 | na |
|  | P_T_<0.05 | 0.02(0.01) | 0.14 | na |
|  | P_T_<0.1 | 0.023(0.01) | 0.086 | na |
|  | P_T_<0.3 | 0.016(0.01) | 0.23 | na |
|  | P_T_<0.5 | 0.021(0.01) | 0.11 | na |
|  | P_T_<0.7 | 0.021(0.01) | 0.12 | na |
|  | P_T_<0.9 | 0.021(0.01) | 0.13 | na |
|  | P_T_<1 | 0.021(0.01) | 0.12 | na |
| 14 y (N=5060) | P_T_<0.001 | -0.005(0.01) | 0.74 | na |
|  | P_T_<0.01 | 0.015(0.01) | 0.3 | na |
|  | P_T_<0.05 | 0.021(0.01) | 0.14 | na |
|  | P_T_<0.1 | 0.024(0.01) | 0.085 | na |
|  | P_T_<0.3 | 0.027(0.01) | 0.053 | na |
|  | P_T_<0.5 | 0.028(0.01) | 0.049 | 0.06 |
|  | P_T_<0.7 | 0.03(0.01) | 0.035 | 0.07 |
|  | P_T_<0.9 | 0.029(0.01) | 0.039 | 0.06 |
|  | P_T_<1 | 0.029(0.01) | 0.038 | 0.07 |
| 17 y (N=4174) | P_T_<0.001 | -0.01(0.02) | 0.52 | na |
|  | P_T_<0.01 | 0.01(0.02) | 0.52 | na |
|  | P_T_<0.05 | 0.012(0.02) | 0.44 | na |
|  | P_T_<0.1 | 0.019(0.02) | 0.23 | na |
|  | P_T_<0.3 | 0.017(0.02) | 0.28 | na |
|  | P_T_<0.5 | 0.017(0.02) | 0.28 | na |
|  | P_T_<0.7 | 0.017(0.02) | 0.27 | na |
|  | P_T_<0.9 | 0.017(0.02) | 0.27 | na |
|  | P_T_<1 | 0.018(0.02) | 0.26 | na |

Polygenic scores for risk-increasing alleles (PGS) in the PGC-ADHD sample were constructed in ALSPAC and then Z-standardised. Rank-transformed SCDC scores were regressed on ADHD-PGS using linear regression, and are shown by PGS threshold (P_T_); Adjusted regression R^2^ (Adj-R^2^) is reported for signals with *p*<0.05; ALSPAC - Avon Longitudinal Study of Parents and Children; ADHD - Attention Deficit Hyperactivity Disorder; na - Not available; PGC - Psychiatric Genomics Consortium; SCDC - SCDC - Social and Communication Disorders Checklist (rank-transformed); y - years
